# Pediatric Idiopathic Bilateral Pneumoparotid

**DOI:** 10.7759/cureus.33621

**Published:** 2023-01-10

**Authors:** Sean Ramsey, Rachel Ely

**Affiliations:** 1 Emergency Department, San Antonio Military Medical Center, San Antonio, USA; 2 Emergency Medicine, Brooke Army Medical Center, San Antonio, USA

**Keywords:** otolaryngology emergency, pediatric, crepitus, mandibular swelling, subcutaneous air, pneumoparotid

## Abstract

A 10-year-old female presented with atraumatic bilateral mandibular swelling. Through imaging and exam she was found to have bilateral pneumoparotid. This is a rare cause of facial swelling that is primarily discussed in otolaryngology literature and relatively unknown in emergency medicine. The presentation can lead to infectious and allergic workups that are unnecessary for the patient. Benign in nature, pneumoparotid is easily diagnosed if an appropriate exam and imaging are completed. Ensuring adequate follow-up and prophylactic treatment with antibiotics are vital to preventing infection.

## Introduction

Facial swelling is a vague and sometimes frustrating chief complaint that is often seen in emergency departments. This is because of the many different etiologies both benign and emergent as the cause. Pneumoparotid is one of the many causes that although rare will likely be seen by many. Differentials associated with this finding range from infectious, traumatic to neoplastic [1] or hypertrophy. Physical exam and history need to be thorough as subtle signs can easily be missed. Subcutaneous emphysema or crepitus are the classic physical exam findings associated with retrograde air into the gland itself. The parotid gland produces serous saliva which moves anterograde through Stensen's duct into the oral cavity. Retrograde movement of air through Stensen's duct is the cause of pneumoparotid [2]. Self-induced pneumoparotid is primarily seen in children with a mean age of 14 while other causes such as glass blowing or wind instruments are seen in primarily adults [3]. Imaging, reassurance, follow-up and antibiotics are the key to treatment. A 10-year-old female presented with atraumatic bilateral facial swelling and mild pain across her mandible. 

## Case presentation

A 10-year-old otherwise healthy female presented with bilateral posterior mandibular facial swelling that began earlier that day without trauma. Her symptoms began while she was playing video games approximately two hours prior to arrival. She did not report any symptoms other than mild bilateral facial pain and a feeling of fullness in her cheeks. Her parents noted that the swelling occurred without any precipitating factor they could identify, denying any recent dental procedure. She denied playing any wind instruments, recent illness with coughs or sneezes, or blowing up any balloons.

Initial physical examination demonstrated mild swelling around the mandibular angles with no erythema, induration or tenderness present. Intraoral examination revealed no signs of dental trauma, mucosal swelling, drainage, posterior erythema or signs of sialolithiasis at the neck of Stensen's duct. Crepitus was felt throughout the patient’s anterior neck extending to the mastoids indicating subcutaneous air.

After extensive conversation with the family CT imaging was deferred for X-ray, secondary to radiation exposure risk. Soft tissue radiographs were obtained to evaluate, demonstrating subcutaneous air extending from the anterior neck and lower face bilaterally. Otolaryngology was consulted, and a diagnosis of pneumoparotid was made. The patient was discharged with Otolaryngology (ENT) follow-up and antibiotics per ENT recommendations to cover oral flora.

## Discussion

Pneumoparotid is classified by free air seen within the parotid glands. Swelling of the parotid glands can be attributed to infection, autoimmune disease, obstruction or benign/malignant growths. Retrograde air into the parotid gland is occurs because of an insufficient Stensen’s duct, normally allowing for flow of saliva into the oral cavity [2]. Insufficiency will go unnoticed until an increase in intraoral pressure causes air to backflow into the parotid gland. Case reports describe causes ranging from holding one's breath, blowing up a balloon to psychiatric tics [2]. In our case there was no identified cause provided in the history from the patient or family. There is no known identifier of an insufficient Stensen’s duct, average measurements of Stensen’s ducts are 5 cm long and 1.7 mm in diameter [4]. The duct itself is located near the second upper molar on both sides, normally it has a small orifice and a mucosal lining and is compressed during increased intraoral pressure stopping insufflation [2]. Postulating with Poiseuille’s Law, R=8ηlπr4, shorter, wider ducts are at higher risk of insufficiency as it would decrease the retrograde resistance needed to overcome the duct itself. Only 54 cases have been reported in literature. Majorly these cases are in the pediatric population (74%), seen in children as young as five, and are linked to psychological stresses or tic disorders. All cases can be linked to an increase in intraoral pressure either self-induced secondary to stress or iatrogenic/idiopathic [2]. Also it can be seen in patients receiving continuous positive airway pressure (CPAP) [2]. Unilateral cases have been reported as well.

Formed during the sixth to 12th week of development [5], parotid glands consist of clusters of acinar cells interspersed with fatty adipocytes and duct cells [6]. Air entering the parotid gland inflates to the level of the acinar cells. Rupturing acinar complexes causes air to track along cervical fascial planes. Crepitus on exam should lead to obtaining further imaging. Simple pneumoparotid shows air within the parotid glands, pneumoparotitis will have inflammatory changes and pain [2], as subtle as mild erythema at Stensen’s duct or purulent discharge with mandibular erythema [7]. Neoplasm cannot be neglected as a possible cause of swollen parotid glands. Neoplasms like Warthin tumors and pleomorphic adenomas have been seen in ages ranging from 16-91 with a mean age of 55 [1], thus decreasing the likelihood of this diagnosis in our 10-year-old patient but not excluding it.

**Figure 1:**
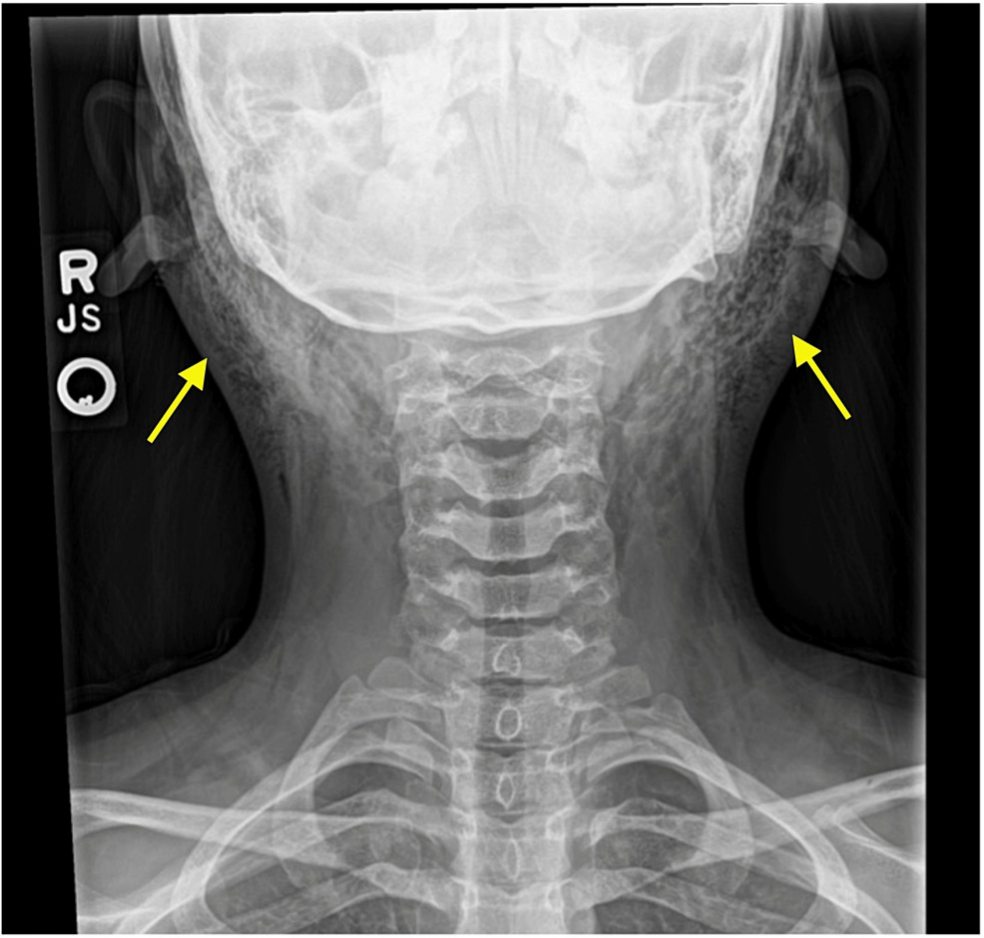
Soft tissue neck X-ray exhibiting subcutaneous emphysema (noted with arrows)

After extensive discussion with family plain X-ray was obtained, foregoing CT secondary to radiation exposure. X-ray showed subcutaneous emphysema along the patient’s anterior neck to the angles of both mandibles (Figure 1). Computed Tomography is the gold standard giving excellent visualization of intraglandular or extraglandular air in subcutaneous tissue [8]. CT with thinner slices is preferable to identify pathology such as stones or abscesses. Ultrasound may be utilized to demonstrate air within the gland [9]. 

Management of pneumoparotid is focused on infection prophylaxis and ensuring resolution of subcutaneous air. Otolaryngology (ENT) consultation and follow up is recommended to ensure resolution. Infection of simple pneumoparotid is a feared complication that can lead to abscess formation or spread of infection through fascial planes. Penicillins are recommended as prophylactic antibiotic treatment to cover for oral flora.

## Conclusions

The patient was discharged with ENT follow-up and antibiotics. During two subsequent follow-up appointments, resolution of subcutaneous air occurred, no infection was reported, and a possible cause was brought to light. The patient reported blowing up a pool toy with minimal pain the day before and blowing into a closed fist just prior to the beginning of her symptoms. Overall, pneumoparotid is a condition that is generally self-induced and self-limited. Physical exam and imaging are pertinent to accurate diagnosis. ENT consultation is recommended to ensure complete resolution and empiric antibiotic of oral flora with penicillins to prevent infection.

## References

[1] Poutoglidis A, Tsetsos N, Sotiroudi S, Fyrmpas G, Poutoglidou F, Vlachtsis K (2020). Parotid gland tumors in northern Greece: a 7-year retrospective study of 207 patients. Otolaryngol Pol.

[2] Gazia F, Freni F, Galletti C (2020). Pneumoparotid and pneumoparotitis: a literary review. Int J Environ Res Public Health.

[3] Yoshida K (2022). Etiology of pneumoparotid: a systematic review. J Clin Med.

[4] Togni L, Mascitti M, Santarelli A, Contaldo M, Romano A, Serpico R, Rubini C (2019). Unusual conditions impairing saliva secretion: developmental anomalies of salivary glands. Front Physiol.

[5] Chason HM, Downs BW (2022). Anatomy, Head and Neck, Parotid Gland. https://www.ncbi.nlm.nih.gov/books/NBK534225/.

[6] Igbokwe CO (2022). Ultrastructure of the parotid salivary gland in the greater cane rats (Thryonomys swinderianus). J Microsc Ultrastruct.

[7] Ghanem M, Brown J, McGurk M (2012). Pneumoparotitis: a diagnostic challenge. Int J Oral Maxillofac Surg.

[8] McGreevy AE, O'Kane AM, McCaul D, Basha SI (2013). Pneumoparotitis: a case report. Head Neck.

[9] Madhavan AA, Carr CM, Carlson ML, Lane JI (2019). Imaging findings related to the valsalva maneuver in head and neck radiology. AJNR Am J Neuroradiol.

